# Auditory evoked-potential abnormalities in a mouse model of 22q11.2 Deletion Syndrome and their interactions with hearing impairment

**DOI:** 10.1038/s41398-024-03218-x

**Published:** 2025-01-08

**Authors:** Chen Lu, Jennifer F. Linden

**Affiliations:** 1 Ear Institute, University College London, London, UK; 2 Department of Neuroscience, Physiology, & Pharmacology, University College London, London, UK

**Keywords:** Neuroscience, Diagnostic markers, Physiology

## Abstract

The 22q11.2 deletion is a risk factor for multiple psychiatric disorders including schizophrenia and also increases vulnerability to middle-ear problems that can cause hearing impairment. Up to 60% of deletion carriers experience hearing impairment and ~30% develop schizophrenia in adulthood. It is not known if these risks interact. Here we used the *Df1/+* mouse model of the 22q11.2 deletion to investigate how hearing impairment might interact with increased genetic vulnerability to psychiatric disease to affect brain function. We measured brain function using cortical auditory evoked potentials (AEPs), which are commonly measured non-invasively in humans. After identifying one of the simplest and best-validated methods for AEP measurement in mice from the diversity of previous approaches, we measured peripheral hearing sensitivity and cortical AEPs in *Df1/+* mice and their WT littermates. We exploited large inter-individual variation in hearing ability among *Df1/+* mice to distinguish effects of genetic background from effects of hearing impairment. Central auditory gain and adaptation were quantified by comparing brainstem activity and cortical AEPs and by analyzing the growth of cortical AEPs with increasing sound level or inter-tone interval duration. We found that level-dependent AEP growth was abnormally large in *Df1/+* mice regardless of hearing impairment, but other AEP measures of central auditory gain and adaptation depended on both genotype and hearing phenotype. Our results demonstrate the relevance of comorbid hearing loss to auditory brain dysfunction in 22q11.2DS and also identify potential biomarkers for psychiatric disease that are robust to hearing impairment.

## Introduction

22q11.2 Deletion Syndrome (22q11.2DS) is the most frequent chromosomal microdeletion syndrome [1] and one of the strongest cytogenetic risk factors for a broad spectrum of psychiatric disorders [2, 3]. For example, nearly 30% of 22q11.2 deletion carriers develop schizophrenia during their lifetime [4–6], and identified symptoms are indistinguishable between schizophrenia patients with the 22q11.2 deletion and those with idiopathic schizophrenia [5, 7]. Insights gained from studying brain abnormalities in 22q11.2DS might therefore have general application to understanding brain abnormalities arising from vulnerability to psychiatric diseases such as schizophrenia. Mouse models of 22q11.2 deletion are also well established, enabling researchers to investigate, at the mechanistic level, what brain abnormalities arise from this genetic risk factor for psychiatric disorders and how experiential risk factors might affect their development [8].

One experiential risk factor for psychiatric disease that may be particularly relevant for 22q11.2DS patients is hearing impairment. Hearing impairment is a recognized experiential risk factor for schizophrenia in the general population [9, 10]. Up to 60% of 22q11.2 deletion carriers have mild to moderate hearing impairment, primarily arising from chronic middle ear inflammation [11, 12]. The possible contribution of hearing impairment to development of psychiatric disease in 22q11.2 carriers has not been studied. However, biomarkers of auditory brain dysfunction, such as abnormalities in cortical auditory evoked potentials (AEPs), are well-documented in 22q11.2DS (and schizophrenia) patients [13, 14]. Surprisingly, the influence of hearing impairment on cortical AEP abnormalities in 22q11.2DS patients has not been systematically examined, despite the prevalence and inter-individual variability of hearing impairment in this population.

Here we investigated whether cortical AEP abnormalities in the *Df1/+* mouse model of 22q11.2DS depend only upon genotype or are affected by hearing phenotype. *Df1/*+ mice have a 1.2 Mb microdeletion homologous to the minimal 1.5 Mb deletion in 22q11.2DS [15] and replicate multiple physiological abnormalities observed in 22q11.2DS patients [16–19], including high inter-individual variation in hearing sensitivity. Like 22q11.2DS patients, *Df1/+* mice are susceptible to chronic middle-ear problems, and up to 60% have mild to moderate hearing impairment in one or both ears [20, 21]. Moreover, previous work has suggested that auditory brain abnormalities in *Df1/+* mice correlate with hearing impairment [22]. Density of parvalbumin-immunoreactive inhibitory interneurons in the auditory cortex was abnormally low in *Df1/+* mice with hearing impairment, and gain of click-evoked cortical AEPs was abnormally high. No significant differences in AEPs were observed between *Df1/+* mice with normal hearing and their WT littermates; however, conclusions were limited by the use of a broadband click stimulus at a single sound level and repetition rate.

In the current study, we first conducted a comprehensive review of methods for cortical AEP measurement in mice, which revealed striking diversity in approaches. Adopting one of the simplest and best-validated strategies, we then investigated cortical AEP abnormalities in *Df1/+* mice in detail using pure tones, varying both tone intensity and inter-tone intervals to study gain and adaptation of auditory brain responses. Our data reveal that some cortical AEP abnormalities in *Df1/+* mice are independent of hearing impairment and others depend on degree of hearing impairment. The results point to specific AEP measures that could be reliable biomarkers for auditory brain abnormalities in 22q11.2DS patients, robust to individual differences in degree of hearing impairment.

## Methods and materials

See Supplementary Information for additional details on acoustic stimulation, experimental procedures, and data pre-processing.

### Animals

Experiments were conducted in 29 *Df1/*+ mice (18 females, 11 males; mean age, 10.3 ± 1.4 weeks) and 22 WT littermates (8 females, 14 males; mean age, 10.2 ± 1.3 weeks). Sample sizes were chosen based on previous related studies of auditory brain abnormalities in *Df1/+* mice [22]. *Df1/*+ mice were originally developed from a 129SvEvBrd X C57BL/6 J background [15] and had been maintained on a C57BL/6 J background for well over 25 generations, through pairing of *Df1/+* males either with WT females from the colony or (at least yearly) with newly acquired C57BL/6 J females from Charles River UK. Mice were raised in standard cages and mouse housing facilities, on a standard 12 h-light/12 h-dark cycle. All methods and experiments were performed in accordance with relevant guidelines and regulations of the UCL Animal Welfare and Ethical Review Body and a UK Home Office project licence approved under the United Kingdom Animals (Scientific Procedures) Act of 1986.

### Recording procedures and measures

Electroencephalographic recordings were performed by an experimenter blind to the genotype of the animal, using procedures similar to those described previously [22]. Briefly, ketamine/medetomidine-anesthetized mice were oriented with the tested ear directed toward the speaker. The opposite ear was blocked with an earplug during recordings to ensure monaural stimulation. We recorded the auditory brainstem response (ABR) differentially from subdermal electrodes at the bulla of the tested ear and the vertex, and the cortical auditory evoked potential (AEP) single-ended from a subdermal electrode over the auditory cortex contralateral to the stimulated ear. The ABR is a sound-evoked signal that occurs within a few milliseconds of stimulus onset and consists of 5 successive waves arising from afferent activity in the auditory nerve and brainstem. The AEP is a later sound-evoked signal consisting of waves thought to originate from the thalamocortical projection (P1), the primary auditory cortex (N1), and higher auditory cortical areas (P2).

We used ABR and AEP recordings to obtain objective measures of hearing threshold, afferent auditory input to the brain, and contralateral cortical responses to auditory input for each ear in each mouse. More specifically, we used click-evoked ABRs to define hearing threshold; amplitude of tone-evoked ABR wave 1 (which arises in the auditory nerve) to quantify afferent input to the auditory brain; amplitudes and latencies of tone-evoked AEP waves to quantify auditory cortical activity; and comparative measures of AEP and/or ABR wave 1 amplitude to measure central auditory gain and adaptation (see Data analysis, below).

### Auditory stimuli

#### Click-evoked ABRs

Stimuli were 50 μs monophasic clicks ranging in sound level from 20 to 90 dB SPL in 5 dB steps, repeated 500 times at each sound level with an inter-onset interval of 50 ms.

#### Tone-evoked ABRs and AEPs

Stimuli were 16 kHz tones (5 ms, cosine-gated ramps) at 80 dB SPL, repeated 1000 times at an inter-tone interval (ITI) of 300 ms.

#### Level-dependent AEP growth functions

Stimuli were 16 kHz tones (5 ms, cosine-gated ramps) at 70, 80, 90, or 100 dB SPL, repeated 1000 times for each sound level with an ITI of 300 ms.

#### Time-dependent AEP growth functions

Stimuli were 16 kHz tones (5 ms, cosine-gated ramps) at 80 dB SPL, repeated 1000 times at ITIs of 200, 250, 300, 350 or 450 ms.

### Data analysis

ABR signals were filtered with a 100–3000 Hz band-pass filter before subtraction (vertex minus bulla electrode) to obtain the differential ABR signal. *Hearing threshold* was defined as the lowest click intensity level eliciting a characteristic ABR wave deflection at least twice as large as the time-dependent standard error in the mean click-evoked ABR waveform. *Tone-evoked ABR wave 1 amplitude and latency* were defined as the amplitude and latency of the peak of ABR wave 1 relative to baseline at stimulus onset; this peak was identified manually for each ear and animal from average ABR waveforms evoked by 16 kHz 80 dB SPL tones.

Three key deflections of the AEP waveform (P1, N1, and P2) were selected from the averaged waveform after removing heartbeat noise [23]. As in previous work [22], P1 was defined as the highest deflection between 15 and 30 ms post stimulus onset; N1 as the lowest deflection between 25 and 60 ms post stimulus onset; and P2 as the highest deflection between 60 and 120 ms post stimulus onset. Amplitude differences P1-N1 or N1-P2 were analyzed to minimize effects of baseline fluctuations on AEP measures. We quantified gain and adaptation of AEPs using three measures. *Central auditory gain* was defined as the ratio between the P1-N1 or N1-P2 AEP amplitude difference and the ABR wave 1 amplitude evoked by the same 16 kHz tone stimulation; this measure quantifies central auditory amplification of peripheral auditory nerve input. *Level-dependent AEP (LDAEP) slope* was defined as the slope of the best-fit linear function relating P1-N1 or N1-P2 AEP amplitude difference (or P1, N1 or P2 latency) to the sound intensity level; this alternate measure of gain was used to quantify central auditory excitability. *Interval-dependent AEP slope* was defined as the slope of the best-fit linear function relating P1-N1 or N1-P2 AEP amplitude difference (or P1, N1 or P2 latency) to the natural logarithm of ITI. We used the natural logarithm because adaptation processes underlying repetition suppression would be expected to decay exponentially with increasing ITI. Interval-dependent AEP slope reflects the dependence of repetition suppression on ITI.

The data were statistically analyzed and graphically visualized using Python. Generalized linear models (GLMs) with a Gaussian distribution assumption were used for the initial assessment of the influence of different mouse-specific variables (genotype, gender, age, hearing threshold of the contralateral ear, and hearing threshold of the ipsilateral ear) on AEP measures of cortical activity recorded over auditory cortex during monaural stimulation of the contralateral ear. We used the Python “glm” function from the “statsmodels” package to fit the GLM equation:

$$\begin{array}{l}{\rm{AEP}}\; {\rm{measure}} \sim {\rm{C}}\left({\rm{genotype}}\right)+{\rm{C}}\left({\rm{gender}}\right)+{\rm{age}}\\\qquad\qquad\qquad\quad\,+\,{\rm{contralateral}}\; {\rm{ear}}\; {\rm{hearing}}\; {\rm{threshold}}\\\qquad\qquad\qquad\quad\,+\,{\rm{ipsilateral}}\; {\rm{ear}}\; {\rm{hearing}}\; {\rm{threshold}}\end{array}$$

where C indicates a categorical rather than quantity variable. Different GLMs were fit for different AEP measures including P1-N1 and N1-P2 amplitude, central auditory gain, level-dependent AEP amplitude change and interval-dependent AEP amplitude change. For each input variable and output AEP measure, the Wald test embedded in the glm function provided a p-value corresponding to the likelihood of observing the GLM weighting for the variable under the null hypothesis that the variable was not predictive of the AEP measure. We concluded that a variable contributed significantly to prediction of an AEP measure if this p-value was less than 0.05.

All other statistical analyses were conducted using non-parametric statistical methods (randomization test, Spearman’s rank correlation test, Mann–Whitney U rank test, Kruskal–Wallis H test with post-hoc Dunn’s test), to ensure results were robust to violations of normality in data distributions. All significance tests were conducted two-tailed with *α* = 0.05, and exact p-values are stated where significant but greater than 0.001.

## Results

### Comprehensive literature review reveals a need for standardized approaches to auditory-evoked potential measurement in mice

**Fig. 1:**
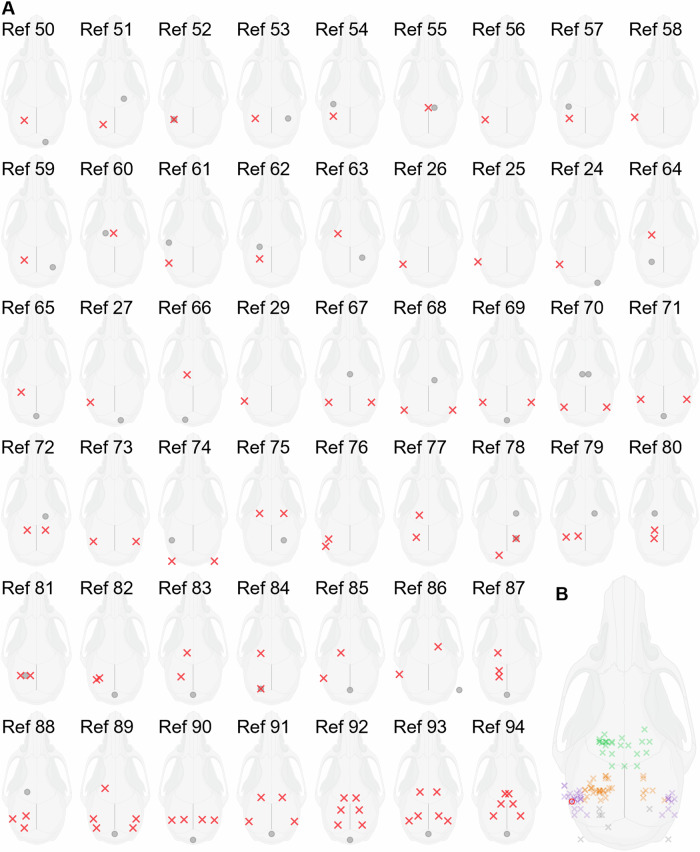
Comprehensive review of electrode placement strategies reveals huge variability in approaches used for measurement of auditory evoked potentials (AEPs) in the mouse. **A** Electrode positions (red cross, positive electrode; gray circle, negative electrode) used for mouse AEP measurements in 50 publications representative of the diversity of approaches in 115 relevant studies identified through a literature search. Unilateral electrode positions are visualised on the left hemisphere to simplify comparison. **B** Summary diagram illustrating the range of positive electrode positions used in previous studies. Positive electrodes were most frequently positioned over the auditory cortex (purple), frontal cortex (green), or hippocampus (orange). Red circle above left auditory cortex indicates the positive electrode location used in the present study during acoustic stimulation of the right ear.

Procedures for cortical AEP recording are relatively well established for human studies, but there is no standardized AEP recording paradigm for mice. To find out how AEP measurement in mice has been performed in the past, we reviewed 115 papers that were obtained from Web of Science using search terms ‘auditory evoked potentials’, ‘event-related potentials’, ‘mouse’, and ‘rodent’. Papers that only covered auditory brainstem responses (sometimes called ‘early AEP’) were excluded. After consolidating the search results to eliminate papers using repeated methods, we still had 50 publications describing different approaches to AEP recording in mice. There was surprisingly little consistency between approaches used by unaffiliated labs. Electrode locations varied from study to study; some used single or symmetric electrode recording from one brain region and others recorded from multiple brain areas simultaneously (Fig. 1A). Clustering all positive electrode locations across studies, we found that in the majority of previous studies, positive electrodes had been placed above auditory cortex, hippocampus and/or frontal areas (Fig. 1B), while reference electrodes were typically located either at the occipital bone or over the frontal/olfactory lobe.

In the absence of a standardized approach to mouse AEP recording, we adopted the simplest strategy compatible with other measurements of intracranial auditory cortical activity correlating with the AEP. Specifically, we placed a single subdermal positive electrode above the auditory cortex contralateral to the monaurally stimulated ear, and a reference electrode over the frontal/olfactory lobe [24–27]. Our definitions of the AEP wave deflections P1, N1 and P2 are consistent with definitions used in those studies [28]. We note also that the same electrode positioning with reversed polarity was used in one of the most detailed characterizations of subcutaneous cortical AEPs in mice to date [29]. Aside from the reversal of polarity, our AEP waveforms were consistent with those recorded by Postal et al. [29], and our P1, N1 and P2 correspond to N23, P35 and N61 from their study.

We used this simple and well-validated approach to AEP measurement in mice to quantify AEP abnormalities in the *Df1/+* mouse model of 22q11.2DS, for comparison with AEP abnormalities observed in 22q11.2DS patients [30]. We focused particularly on a question that has been largely overlooked in previous studies of 22q11.2DS patients and 22q11.2DS model mice: are AEP abnormalities in *Df1/+* mice related to hearing impairment, a common comorbidity of 22q11.2DS?

### High inter-individual and inter-ear variation in hearing sensitivity in *Df1*/+ mice necessitates ear-by-ear analysis of effects of hearing impairment

**Fig. 2:**
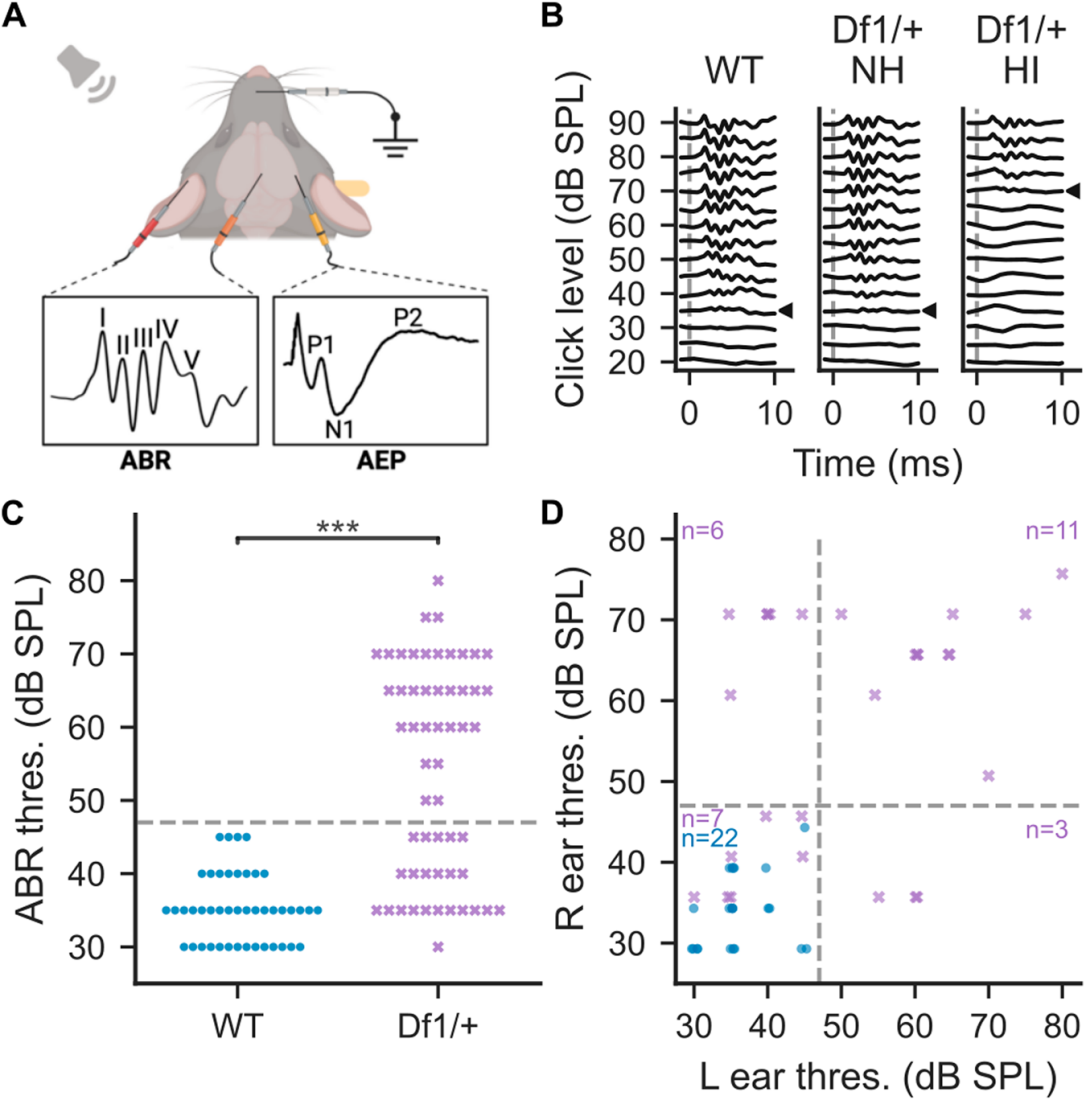
Experimental setup enables ear-by-ear quantification of peripheral hearing sensitivity. **A** ABRs and AEPs were measured with subdermal electrodes in anesthetized mice. **B** Hearing sensitivity of each ear was quantified by identifying the click-evoked ABR threshold, i.e. the minimum click intensity eliciting a detectable ABR waveform. Three examples are shown, with click-evoked ABR thresholds indicated by arrowheads: left (WT) 35 dB SPL, middle (*Df1/*+ with normal hearing) 35 dB SPL, right (*Df1/*+ with hearing impairment) 70 dB SPL. Dashed vertical line indicates click onset. **C** Distributions of click-evoked ABR thresholds in WT ears (blue circles) and *Df1/*+ ears (purple crosses). Ears with hearing impairment were defined as those with click-evoked ABR thresholds >2.5 standard deviations above the mean WT threshold (i.e., above 47 dB SPL; dashed line). **D** Comparison of click-evoked ABR thresholds in the right and left ears; conventions as in **C**.

We first confirmed previous findings that *Df1/+* mice replicate the high inter-individual and inter-ear variation in hearing sensitivity also observed in 22q11.2DS patients [12, 20, 22]. We quantified peripheral hearing sensitivity of each ear in each mouse using the click-evoked ABR (see Methods and Materials). ABRs were measured with subdermal electrodes (Fig. 2A) during presentations of click stimuli varying in sound level from 20 dB to 90 dB SPL. The hearing threshold of an ear was defined as the lowest click intensity level eliciting a significant ABR wave deflection (Fig. 2B).

Hearing thresholds of *Df1/*+ ears were bimodally distributed (Fig. 2C), as demonstrated previously [20, 22]. On average, hearing thresholds for *Df1/*+ ears were significantly elevated compared to those from their WT littermates (*Df1/*+, 54.02 ± 14.41 dB SPL from 56 ears, WT, 35.23 ± 4.64 dB SPL from 44 ears; Wilcoxon rank-sum test, p < 0.001), but many *Df1*/+ ears had WT-like hearing thresholds. Here, we defined an ear as exhibiting hearing impairment if its hearing threshold was more than 2.5 standard deviations above the mean hearing threshold for WT ears (47 dB SPL). Then we grouped *Df1/*+ ears into “normal hearing” (*Df1/*+ NH, 23 ears) and “hearing impaired” (*Df1/*+ HI, 33 ears).

Notably, hearing impairment in one ear of a *Df1/+* mouse did not predict hearing impairment in the other. *Df1/+* mice often had hearing impairment in only one ear, and there was no significant correlation between the hearing thresholds of the two ears in *Df1/+* mice (Spearman’s rank correlation, ρ = 0.31, p = 0.12). Among all *Df1/*+ mice tested in both ears, 26% (7/27) had normal hearing in both ears, 33% (9/27) had unilateral hearing impairment (6 in right ear, 3 in left ear), and 41% (11/27) had bilateral hearing impairment (Fig. 2D). There were no gender differences in hearing thresholds for either *Df1/+* and WT mice (data not shown; see [20]).

Based on the evidence that there was no correlation in hearing thresholds between ears, we adopted individual ears rather than mice as the unit for analysis. We confirmed this approach was valid for analysis of AEP measures using generalized linear models (GLMs) and randomization tests. Specifically, we used GLMs (see Methods and materials) to investigate how our AEP measures of cortical activity in each mouse, recorded over auditory cortex in each hemisphere during monaural stimulation of the contralateral ear, depended on the hearing threshold of the contralateral ear, the hearing threshold of the ipsilateral ear, and the genotype, gender and age of the mouse. Results of the GLM analyses (Table 1) indicated that the only consistently predictive variables were the hearing threshold of the contralateral ear and the genotype of the mouse; the gender of the mouse was also predictive but for only one of the AEP measures analyzed. Importantly, the hearing threshold of the ipsilateral ear did not contribute significantly to prediction of any of the AEP measures. We corroborated this result using randomization tests, which demonstrated that correlation between paired AEP measures from stimulation of different ears in the same animal was not significantly different from correlation between independent AEP measures from different animals (Supplementary Fig. 1). Within-animal correlation fell within the 95% confidence interval for between-animal correlation for all AEP measures and for both P1-N1 and N1-P2 waves.

**Table 1:** Generalized linear model (GLM) analyses of variables affecting AEP measures.

	Genotype	Age	Gender	Threshold of contralateral ear	Threshold of ipsilateral ear
P1-N1 amplitude	<0.001***	0.698	0.632	0.015*	0.151
N1-P2 amplitude	0.005**	0.726	0.894	0.182	0.107
P1-N1 central gain	0.045*	0.952	0.463	0.001**	0.215
N1-P2 central gain	0.139	0.750	0.424	<0.001***	0.236
P1-N1 level dependence	0.003**	0.634	0.001**	0.179	0.362
N1-P2 level dependence	0.115	0.429	0.059	0.209	0.229
P1-N1 interval dependence	<0.001***	0.852	0.978	0.037*	0.709
N1-P2 interval dependence	0.001**	0.133	0.294	0.035*	0.706

For each of the AEP measures indicated in rows, a GLM analysis was performed to assess the influence of animal-specific variables shown in columns. P-values indicate the likelihood of observing the GLM weighting for the variable under the null hypothesis that it was not predictive of the AEP measure. Significant p-values are shown in bold with asterisks indicating *p < 0.05, **p < 0.01 or ***p < 0.001. Each mouse contributed two AEP recordings to the GLM analysis for each AEP measure, one for each auditory cortical hemisphere and the contralateral, monaurally stimulated ear. Genotype and threshold of the contralateral ear emerged as key variables with significant influence on multiple AEP measures. Gender was a significant variable only for the P1-N1 level-dependent AEP measure. There was no significant influence of the threshold of the ipsilateral ear on any of the AEP measures. Therefore, in further analyses of each AEP measure, we treated AEP recordings corresponding to contralateral ears rather than mice as the unit of analysis.

We therefore performed further analyses of our cortical AEP measures grouping AEP recordings by the threshold of the contralateral ear and the genotype of the mouse, with supplemental analysis of gender differences where indicated by the GLM results.

### Tone-evoked auditory cortical potentials and brainstem responses confirm increased central auditory gain in *Df1*/+ mice with hearing impairment

Central auditory gain (excitability) can be quantified by comparing the amplitude of central auditory evoked potentials to the amplitude of ABR wave 1, which reflects auditory nerve input to the brain [31]. We recorded tone-evoked cortical AEPs simultaneously with ABRs to test the hypothesis that central auditory gain is elevated in *Df1/+* mice with hearing impairment. A previous study had concluded that *Df1/+* mice with hearing impairment exhibited increased central auditory gain, based on the observation that the ratio of click-evoked AEP to ABR magnitude was abnormally elevated for *Df1/+* ears with hearing impairment [22]. This conclusion is consistent with substantial literature demonstrating that hearing impairment increases the excitability of neurons throughout the ascending central auditory system [32]. However, it is possible that alterations in click-evoked AEP/ABR ratio with hearing impairment could arise simply from the different frequency sensitivities of the ABR versus AEP generators (for example, if hearing impairment primarily affected high sound frequencies, which drive the ABR more strongly than the AEP), not from changes in central auditory gain.

**Fig. 3:**
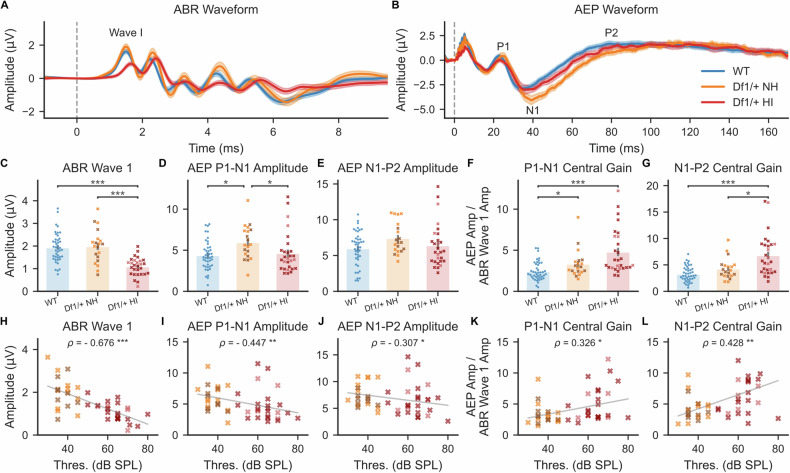
Abnormalities in central auditory gain in *Df1/+* mice are evident even during stimulation of ears with normal hearing but are most pronounced for stimulation of ears with hearing impairment. Central auditory gain (AEP/ABR amplitude ratio) was elevated in *Df1/*+ mice for a 16 kHz tone stimulus, and positively correlated with click-evoked ABR threshold. **A**, **B** Averaged tone-evoked ABR waveforms measured between tested ear bulla and vertex (**A**) and AEP waveforms measured from the contralateral hemisphere (**B**) during monaural stimulation of WT NH, *Df1/*+ NH, and *Df1/*+ HI ears with 80 dB SPL, 16 kHz tones. Dashed vertical line indicates stimulus onset. **C** Tone-evoked amplitude of ABR wave 1 was significantly reduced for *Df1/+* HI ears versus *Df1/+* NH or WT NH ears. **D**, **E** However, cortical AEP waves measured contralateral to the stimulated ear showed a different pattern. Amplitude of the AEP P1-N1 complex was largest for stimulation of *Df1/+* NH ears (**D**); a similar trend was evident for the AEP N1-P2 complex (**E**). **F**, **G** Central gain (tone-evoked contralateral AEP amplitude normalized by tone-evoked ABR wave 1 amplitude) was abnormally elevated for the P1-N1 complex during stimulation of *Df1/+* NH ears (**F**), and for both the P1-N1 and N1-P2 complexes during stimulation of *Df1/+* HI ears (**F**, **G**). **H**–**J** Amplitude of ipsilateral ABR wave 1 (**H**), contralateral AEP P1-N1 complex (**I**), or contralateral AEP N1-P2 complex (**J**) evoked by an 80 dB SPL, 16 kHz tone, versus click-evoked ABR threshold for the stimulated ear. Negative correlation with hearing threshold in the stimulated ear was weaker for tone-evoked AEP than ABR. **K**, **L** Central gain of tone-evoked responses was positively correlated with click-evoked ABR threshold for both the P1-N1 complex (**K**) and N1-P2 complex (**L**). Each animal typically contributed two data points, one for each ear, with NH or HI status determined separately for each ear based on click-evoked ABR thresholds. Hearing sensitivity in the opposite (unstimulated) ear is indicated by the darkness of symbols: darker symbols indicate cases in which the opposite ear was hearing impaired. Plot conventions: blue circles, WT; yellow cross, *Df1/*+ NH with normal hearing in the unstimulated ear; brown cross, *Df1/*+ NH with hearing impairment in the unstimulated ear; red cross, *Df1/*+ HI with normal hearing in the unstimulated ear; dark red cross, *Df1/*+ HI with hearing impairment in the unstimulated ear; gray solid line, 2D least-mean-squares best-fit line to the *Df1/*+ data; asterisks, significance threshold (*p < 0.05, **p < 0.01, ***p < 0.001) for Dunn’s post-hoc tests (**C**–**G**) or Spearman’s rank correlation tests (**H**–**L**).

To rule out this possible alternative explanation, we investigated the relationship between hearing impairment and the AEP/ABR ratio measure of central auditory gain using 80 dB SPL, 16 kHz tones instead of clicks, and confirmed that elevated central auditory gain is evident in tone-evoked brain activity in *Df1/+* mice. Importantly for this analysis, clicks were used only for categorizing *Df1/+* ears as normal hearing or hearing impaired based on click-evoked ABR threshold, not for analysis of central auditory gain. As expected given the click-evoked ABR thresholds, the amplitude of ABR wave 1 evoked by a 80 dB SPL, 16 kHz tone was much smaller for *Df1/*+ HI ears than for *Df1*/+ NH or WT ears (Fig. 3A, C; Kruskal–Wallis test, p < 0.001, post-hoc tests, p_*Df1/*+ NH-*Df1/*+ HI_ < 0.001, p_*Df1/*+ NH-WT_ = 0.98, p_*Df1/*+ HI-WT_ < 0.001). However, there was no corresponding drop in the amplitude of cortical AEP waveforms evoked at *Df1/+* HI ears compared to WT ears, and tone stimulation of *Df1/+* NH ears produced AEPs with *larger* P1-N1 amplitude than for WT ears (Fig. 3B, D and E; Kruskal–Wallis test, P1-N1, p = 0.0015, post-hoc tests, p_*Df1/*+ NH-*Df1/*+ HI_ = 0.017, p_*Df1/*+ NH-WT_ = 0.017, p_*Df1/*+ HI-WT_ = 0.79; N1-P2, p = 0.034, post-hoc tests, p_*Df1/*+ NH-*Df1/*+ HI_ = 0.20, p_*Df1/*+ NH-WT_ = 0.20, p_*Df1/*+ HI-WT_ = 0.83). The gain of tone-evoked auditory brain activity was elevated in *Df1/+* mice, not only during stimulation of *Df1/+* HI ears but also (for the P1-N1 complex) during stimulation of *Df1/+* NH ears (Fig. 3F, G; Kruskal–Wallis tests, P1-N1: p < 0.001, post-hoc tests, p_*Df1/*+ NH-*Df1/*+ HI_ = 0.083, p_*Df1/*+ NH-WT_ = 0.043, p_*Df1/*+ HI-WT_ < 0.001; N1-P2, p < 0.001, post-hoc tests, p_*Df1/*+ NH-*Df1/*+ HI_ = 0.037, p_*Df1/*+ NH-WT_ = 0.11, p_*Df1/*+ HI-WT_ < 0.001).

Further analysis demonstrated that central auditory gain in *Df1/+* mice was positively correlated with the degree of hearing impairment in the stimulated ear. We examined the relationship between click-evoked hearing thresholds and tone-evoked ABR wave 1 amplitude, AEP P1-N1 or N1-P2 amplitude, or AEP/ABR ratio measures of central auditory gain in *Df1/+* mice. Unsurprisingly, the amplitude of tone-evoked ABR wave 1 was negatively correlated with click-evoked hearing threshold in the stimulated ear (Fig. 3H; Spearman’s rank correlation test, ρ = −0.68, p < 0.001). Amplitudes of tone-evoked P1-N1 and N1-P2 complexes measured over the contralateral auditory cortex were also negatively correlated with click-evoked hearing thresholds (Fig. 3H, I; P1-N1 complex: ρ = −0.45, p = 0.0015; N1-P2 complex: ρ = −0.31, p = 0.034). However, there were robust positive correlations between tone-evoked central auditory gain measures (AEP/ABR ratios) and click-evoked hearing thresholds, for both the P1-N1 and N1-P2 complex (Fig. 3K, L; P1-N1 complex AEP/ABR: ρ = 0.33, p = 0.024; N1-P2 complex AEP/ABR: ρ = 0.43, p = 0.0024).

Interestingly, we also found that latencies of late tone-evoked AEP waves were longer in *Df1/+* than WT mice, regardless of hearing impairment (Supplementary Fig. 2). Tone stimulation of *Df1/*+ ears with or without hearing impairment evoked N1 and P2 waves with significantly longer latencies than those evoked in WT mice, while P1 wave latencies were not significantly different (Supplementary Fig. 2A–C). There was no significant correlation between AEP wave latencies in *Df1/+* mice and the click-evoked hearing threshold in the stimulated ear for any of the three AEP waves (Supplementary Fig. 2D–F). Thus, tone-evoked N1 and P2 wave latencies were significantly longer for *Df1/+* than WT mice, even when the stimulated *Df1/+* ears had normal hearing thresholds.

### Higher level-dependent AEP growth in *Df1*/+ mice, with or without hearing impairment

Another measure commonly used to investigate changes in central auditory excitability is growth of cortical AEP amplitude with increasing sound level. A previous study reported steeper level-dependent AEP growth in the *Df(h22q11)/+* mouse model of 22q11.2DS than in WT mice during stimulation with white noise bursts [24]. Here, we used 16 kHz tones to compare level-dependent AEP (LDAEP) growth functions not only between *Df1/+* mice and their WT littermates but also between *Df1/+* mice with or without hearing impairment in the stimulated ear.

**Fig. 4:**
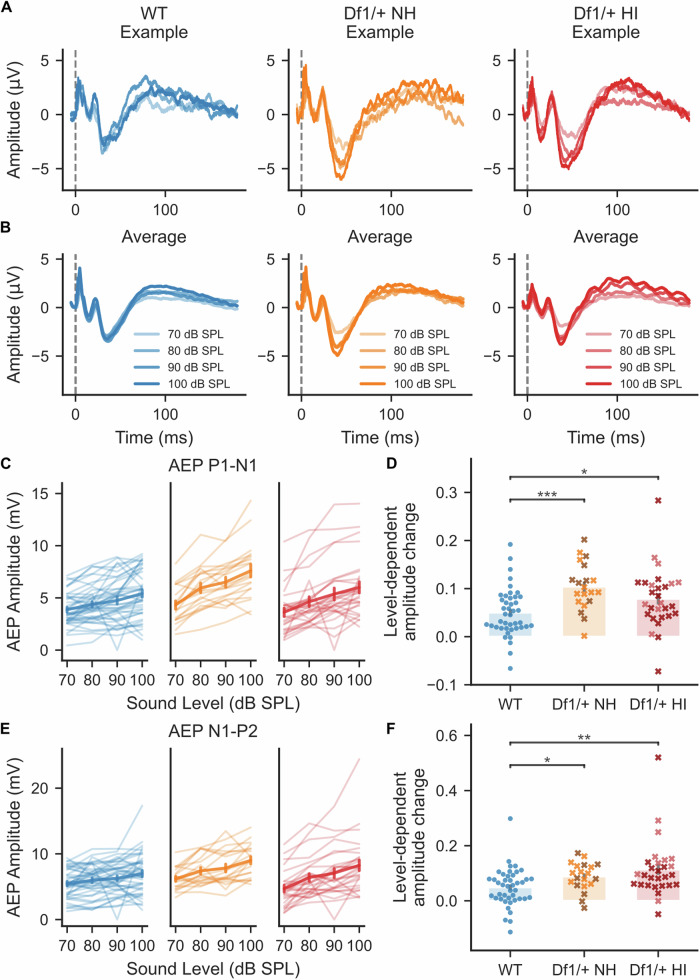
Abnormalities in level-dependent AEP growth in *Df1/+* mice are robust to hearing impairment in the stimulated ear. Growth of AEP amplitude with increasing tone intensity was steeper in *Df1/+* mice than WT mice, even for stimulation of *Df1/+* ears with normal hearing. **A**, **B** Example AEP waveforms (**A**) and average AEP waveforms (**B**) evoked by a 16 kHz tone presented at different sound intensity levels. **C**, **E** AEP amplitudes for the tone-evoked P1-N1 complex (**C**) and N1-P2 complex (**E**) grew more steeply with increasing sound level for *Df1/+* mice than WT mice, regardless of the hearing status for the stimulated *Df1/+* ear. Thin lines represent recordings from contralateral hemispheres of individual ears; thick line with error bars shows mean ± SEM. **D**, **F** For both the P1-N1 complex (**D**) and the N1-P2 complex (**F**), slopes of level-dependent AEP growth functions were higher for stimulation of either *Df1/*+ NH or *Df1/*+ HI ears than for WT NH ears. Plot conventions as in Fig. 3C–G.

We found that tone-evoked cortical AEP wave amplitudes grew more steeply with increasing sound level in *Df1/*+ mice than WT mice, regardless of whether the stimulated *Df1/+* ear was normal hearing or hearing impaired (Fig. 4). This effect was clearly evident both in example AEP waveform recordings and in group averages (Fig. 4A, B). We calculated the AEP amplitude as a linear function of sound level and used the function slope to represent the level-dependence of the AEP (Fig. 4C, E). For both the P1-N1 and N1-P2 complexes, the LDAEP amplitude change was larger in *Df1/+* mice than in their WT littermates (Fig. 4D, F; P1-N1: Kruskal–Wallis test, p < 0.001, post-hoc tests, p_*Df1/*+ NH-*Df1/*+ HI_ = 0.092, p_*Df1/*+ NH-WT_ < 0.001, p_*Df1/*+ HI-WT_ = 0.040; N1-P2: Kruskal–Wallis test, p = 0.0020, post-hoc tests, p_*Df1/*+ NH-*Df1/*+ HI_ = 0.75, p_*Df1/*+ NH-WT_ = 0.022, p_*Df1/*+ HI-WT_ = 0.0042). P1-N1 LDAEP was larger overall in female than male mice, but the significant effect of genotype on P1-N1 LDAEP was preserved in GLM analysis (Table 1) and also evident as a trend in comparisons between genotypes within gender (Mann-Whitney U rank test, p_male WT-*Df1/+*_ = 0.034, p_female WT-*Df1/+*_=0.061). There were no significant differences in LDAEP amplitude change for data obtained during stimulation of *Df1/+* NH versus *Df1/+* HI ears, suggesting that level-dependent AEP amplitude change could be a biomarker for central auditory abnormalities in *Df1/+* mice that are robust to hearing impairment.

Level-dependent changes in AEP wave latencies were less robust as a potential biomarker for abnormalities in *Df1/+* mice (Supplementary Fig. 3). AEP P1 and N1 latencies decreased more steeply with increasing tone intensity level in *Df1/+* mice compared to their WT littermates, but effects were not consistently significant for the two waves across *Df1/+* NH and *Df1/+* HI ears. For P2, there were no significant differences between the groups in the relationship between wave latency and sound level.

### Altered repetition suppression in *Df1/*+ mice with normal hearing

Sound repetition typically causes suppression of auditory evoked activity, and this suppression is stronger at shorter inter-stimulus intervals. The dependence of cortical AEP amplitude on inter-stimulus interval is therefore a measure of repetition suppression; more generally, it is a potential biomarker for abnormalities in central auditory adaptation. A recent study of auditory processing endophenotypes in 22q11.2DS patients showed that tone-evoked AEP amplitudes were more strongly dependent on inter-tone interval (ITI) in patients without psychotic symptoms than in either patients with psychotic symptoms or neurotypical controls [30]. We wondered if similar abnormalities might be evident in *Df1/+* mice, and if so, how these abnormalities might relate to hearing impairment. We measured the sensitivity of the AEP to changes in the temporal structure of the stimulus using 16 kHz, 80 dB SPL tones presented at varying ITIs.

At first we used ITIs varying from 200 ms to 350 ms (Supplementary Fig. 4), and observed that AEP amplitude increased with ITI to a plateau in many cases. The turning point for the plateau in WT and *Df1/*+ HI data was between 300 and 350 ms, while the turning point for *Df1/*+ NH data was not clear and could have been at longer ITIs. Therefore, we extended the range of ITIs to 200–450 ms in further experiments.

**Fig. 5:**
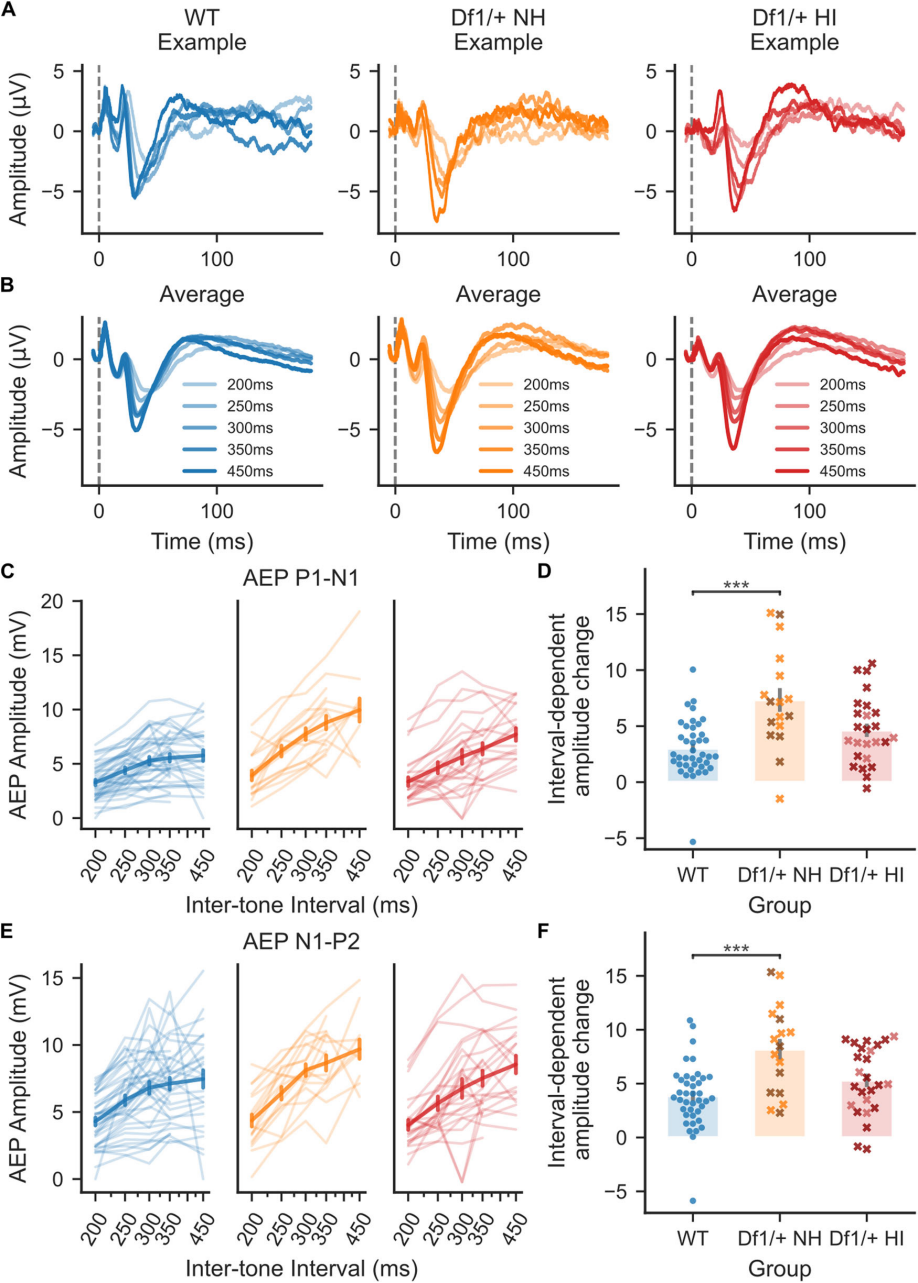
Abnormalities in repetition suppression in *Df1/+* mice are evident during stimulation of ears with normal hearing. Growth of AEP amplitude with increasing inter-tone interval (ITI) was steeper for stimulation of *Df1/*+ NH ears than for WT ears. **A**, **B** Example AEP waveforms (**A**) and average AEP waveforms (**B**) evoked by 80 dB SPL, 16 kHz tones presented at different ITIs. **C**, **E** AEP amplitudes for the tone-evoked P1-N1 complex (**C**) and N1-P2 complex (**E**) grew more steeply with increasing ITI in *Df1/+* mice than WT mice, particularly at longer ITIs. The natural logarithm of ITI was used for analysis to reflect the assumption of exponential decay with increasing ITI in adaptation processes underlying repetition suppression. Note the apparent plateau in AEP amplitude at 300–450 ms ITIs in WT data, and continued growth with ITI in *Df1/+* data, especially for stimulation of *Df1/+* ears with normal hearing. **D**, **F** For both the P1-N1 complex (**D**) and the N1-P2 complex (**F**), slopes of interval-dependent AEP growth functions were higher for stimulation of *Df1/*+ NH ears than WT ears. A similar trend, but no significant difference, was observed for comparison of interval-dependent AEP growth-function slopes for stimulation of *Df1/+* HI ears versus WT ears; note however that interpretation of this latter result is complicated by the fact that the fixed-intensity tone stimulus was closer to hearing threshold for *Df1/+* HI ears than WT (NH) ears. Plot conventions as in Fig. 4; natural logarithmic x-axis in **C** and **E**.

We found that repetition suppression was significantly weaker at longer ITIs for *Df1/+* NH recordings than for either WT or *Df1/+* HI recordings (Fig. 5). To quantify the dependence of repetition suppression on ITI, we calculated the best-fit linear function relating AEP amplitude to the natural logarithm of ITI duration (Fig. 5C, E). We used the slope of this function as a measure of interval-dependent AEP growth (Fig. 5C–F). For both the P1-N1 and N1-P2 complexes, interval-dependent AEP growth was abnormally large in *Df1/+* NH recordings, relative to either WT data or *Df1/+* HI data (Fig. 5D, F; P1-N1: Kruskal–Wallis test, p < 0.001, post-hoc tests, p_*Df1/*+ NH-*Df1/*+ HI_ = 0.060, p_*Df1/*+ NH-WT_ < 0.001, p_*Df1/*+ HI-WT_ = 0.069; N1-P2: p = 0.0010, post-hoc tests, p_*Df1/*+ NH-*Df1/*+ HI_ = 0.10, p_*Df1/*+ NH-WT_ < 0.001, p_*Df1/*+ HI-WT_ = 0.10). Larger interval-dependent AEP growth in the *Df1/+* NH group than in the WT and *Df1/+* HI groups was also evident when data were normalized to AEP amplitudes at 350 ms (the longest ITI used in all experiments) to rule out effects of group differences in overall AEP amplitudes (Supplementary Fig. 5). This result indicates that central auditory adaptation to repeated tones differs between *Df1/+* NH and WT mice, and suggests that hearing impairment in *Df1/+* HI mice may either mask or counteract this abnormality.

We also examined the relationship between AEP wave latency and ITI, comparing *Df1/+* NH, *Df1/+* HI, and WT data. For the P1, N1, and P2 waves, latency decreased similarly with increasing ITIs in all groups (Supplementary Fig. 6). There were no significant group differences in the relationship between AEP wave latency and ITI for P1 or P2, and for N1, a weak group difference was not robust to post-hoc testing.

## Discussion

Our results reveal multiple abnormalities in cortical auditory evoked potentials (AEPs) in the *Df1/+* mouse model of 22q11.2DS and demonstrate that some of these abnormalities are strongly influenced by mild to moderate hearing impairment, a common co-morbidity of 22q11.2DS. Using one of the best-validated of the surprisingly diverse and unstandardized approaches to mouse AEP measurement in the literature, we measured tone-evoked cortical AEPs along with ABRs in both *Df1/+* mice and their WT littermates. We quantified hearing thresholds ear-by-ear in every mouse using click-evoked ABR thresholds, so that we could exploit the high inter-individual and inter-ear variation in hearing sensitivity typical of *Df1/+* mice (and 22q11.2DS patients) to probe the relationship between AEP abnormalities and hearing impairment.

We found 3 key AEP abnormalities in *Df1/+* mice, which exhibited differing relationships to hearing impairment. Tone-evoked central auditory gain (AEP amplitude normalized by ABR amplitude) was elevated primarily in *Df1/+* mice with hearing impairment, and strongly correlated with the level of hearing impairment in the stimulated ear. Another measure of central auditory excitability—growth of cortical AEP amplitude with increasing sound level—was robustly elevated in all *Df1/+* mice, even when hearing was normal in the stimulated ear. Finally, growth of AEP amplitude with increasing inter-tone interval, a measure of central auditory adaptation and recovery from repetition suppression, was abnormal in *Df1/+* mice only when hearing was normal in the tested ear. In summary, these results show that *Df1/+* mice have abnormalities in central auditory gain (AEP/ABR ratio) that are exacerbated by hearing impairment; abnormalities in central auditory excitability (level-dependent AEP amplitude growth) that are robust to hearing impairment; and abnormalities in auditory adaptation (interval-dependent AEP amplitude growth) that are either counteracted or masked by hearing impairment.

### Implications of results

Two points need to be kept in mind when considering the implications of our results.

First: the unusual nature of hearing impairment in *Df1/+* mice (and 22q11.2DS patients) means that ear-by-ear analysis of hearing sensitivity is essential, and monaural auditory stimulation for AEP measurement is advisable to simplify data analysis and increase statistical power. Like 22q11.2DS patients, *Df1/+* mice are susceptible to chronic middle ear inflammation that can cause mild to moderate hearing impairment in both ears or in only one ear [20, 22]. Hearing impairment in our cohort of *Df1/+* mice was frequently unilateral, and there was minimal correlation in hearing thresholds between the two ears. Since auditory cortical AEPs are most strongly driven by contralateral ear stimulation (see for example Postal et al. [29]), we reasoned that effects of hearing impairment on central auditory function in *Df1/+* mice would best be investigated by recording AEPs over each cortical hemisphere in each animal during monaural stimulation of the contralateral ear. Subsequent GLM analyses showed that our cortical AEP measures depended on the hearing threshold only of the contralateral (stimulated) ear, not the ipsilateral (unstimulated) ear. Furthermore, correlation between paired AEP measures from stimulation of different ears in the same animal was not significantly different from correlation between independent AEP measures from different animals. These results simplified further analyses by enabling us to treat AEP recordings corresponding to stimulated ears rather than mice as the unit of analysis. However, it is likely that AEPs measured during binaural stimulation would depend on hearing thresholds in both ears, increasing the variability of experimental results and the complexity of data analysis. Therefore, one implication of this work in *Df1/+* mice is that AEP abnormalities in 22q11.2DS patients might best be identified using monaural auditory stimulation paradigms, rather than the usual binaural stimulation paradigms (e.g., [13, 30, 33, 34]).

Second: high inter-individual and inter-ear variation in hearing sensitivity among *Df1/+* mice (and 22q11.2DS patients) complicates interpretation of AEP abnormalities. As in most AEP studies of 22q11.2DS patients [13, 30, 33, 34], we presented exactly the same set of auditory stimuli to each subject (and each ear). We did not adjust the sound level of the stimuli to be constant relative to the hearing threshold of the stimulated ear, to avoid the use of extremely loud stimuli for stimulation of ears with moderate hearing impairment (which would have undermined our ability to ensure monaural stimulation using an earplug in the opposite ear). However, the use of fixed-intensity stimuli meant that inevitably, weaker auditory nerve signals were evoked in ears with hearing impairment than in ears with normal hearing. The AEP/ABR ratio measure of central auditory gain attempts to normalize for differences in auditory nerve activity during stimulation of *Df1/+* HI versus *Df1/+* NH or WT ears, making comparisons across all three groups more valid. However, differences in auditory nerve activity are not fully taken into account by measures of level-dependent and interval-dependent AEP growth, complicating comparisons between hearing-impaired and normal-hearing data groups. Therefore, the most compelling abnormalities are those revealed in *Df1/+* NH versus WT comparisons: higher level-dependent and interval-dependent AEP amplitude growth.

### Biomarkers for central auditory processing abnormalities in 22q11.2DS

Interestingly, similar abnormalities in level-dependent and interval-dependent AEP growth have been mentioned previously in studies of 22q11.2DS models or patients, although possible interactions with hearing impairment have never before been explored.

In the *Df(h22q11)/+* mouse model of 22q11.2DS, Didriksen et al. [24] reported abnormally high LDAEP growth compared to WT littermates, as we report here for *Df1/+* mice either with or without hearing impairment. Notably, hearing thresholds were assessed ear-by-ear using ABR in a separate cohort of these mice and were not significantly different between *Df(h22q11)/+* and WT animals, although unusually high for WT mice [24]. In human 22q11.2 deletion carriers, LDAEP growth has not been examined, but multiple studies have reported elevated N1 amplitude in 22q11.2DS patients [30, 34, 35]. While these findings are sometimes difficult to interpret where normal hearing has been confirmed only by self-report, the data suggest that auditory cortical responses to loud sounds may be abnormally large in 22q11.2DS patients as well as in *Df1/+* and *Df(h22q11)/+* mice.

Repetition suppression abnormalities analogous to those we report in *Df1/+* mice have also been observed in 22q11.2DS patients. In 22q11.2DS patients with confirmed normal hearing and without psychotic symptoms, Francisco et al. [30] found abnormally large growth in N1 amplitude with increasing inter-tone interval (ITI), just as we observed in *Df1/+* mice during stimulation of ears with normal hearing. Participants were included in the Francisco et al. [30] study only if they passed an audiometric test to ensure that hearing thresholds were no more than 25 dB above normal in both ears. Interestingly, there were no significant differences in N1 amplitude at long ITIs between 22q11.2DS patients *with* psychotic symptoms and neurotypical age-matched controls. Since reductions in N1 amplitude have been reliably demonstrated in both schizophrenia patients and first-degree relatives of individuals with schizophrenia [36, 37], Francisco et al. [30] suggested that the apparently normal N1 amplitudes in 22q11.2DS patients with psychotic symptoms might be masking opposing abnormalities: abnormal elevation of N1 amplitude at long ITIs arising from the 22q11.2 deletion, and abnormal reduction of N1 amplitude arising from psychosis symptomatology [30]. Similarly, in our study, it is possible that interval-dependent AEP growth was abnormally high during stimulation of *Df1/+* ears with normal hearing but not *Df1/+* ears with hearing impairment because reduction in hearing sensitivity masked central auditory abnormalities in repetition suppression caused by the *Df1* deletion.

In summary, together with our own our data from *Df1/+* mice, the Didriksen et al. [24] and Francisco et al. [30] studies suggest that both level-dependent and interval-dependent AEP growth are abnormally elevated in 22q11.2DS, at least in the absence of hearing impairment. Our work further demonstrates that elevated LDAEP growth may be a particularly useful biomarker for central auditory processing abnormalities in 22q11.2DS, because this AEP abnormality is robust to hearing impairment in the stimulated ear.

### Mechanisms contributing to AEP abnormalities

What mechanisms might underlie the AEP abnormalities we observed in *Df1/+* mice? From a systems-level perspective, increased central auditory gain and LDAEP growth indicate increased excitability in subcortical auditory structures and/or the auditory cortex. The interval-dependent AEP results reveal normal adaptation at <250 ms inter-tone intervals in *Df1/+* mice but reduced adaptation (larger AEP) at >300 ms inter-tone intervals, perhaps reflecting normal subcortical adaptation processes but abnormally weak damping of recurrent excitation in higher auditory cortex. Hearing impairment is already known to cause increased excitability and decreased inhibitory synaptic transmission in the auditory cortex [32, 38–41]. Moreover, previous work has found that parvalbumin-expressing (PV) inhibitory interneurons, which play a critical role in auditory cortical gain control, adaptation and recurrent excitation [42], are abnormally sparse in the auditory cortex of *Df1/+* mice—and this PV interneuron deficit correlates with degree of hearing impairment [22]. More generally, deficits in inhibitory signaling have been reported in the prefrontal cortex and hippocampus in other mouse models of 22q11.2DS [43, 44], and human 22q11.2DS cerebral cortical organoids have been found to display increased spontaneous firing [45]. It is possible that both the 22q11.2 deletion and hearing impairment alter auditory cortical excitability and adaptation in similar ways, and that auditory brain abnormalities in 22q11.2DS arise from interactions between genetic vulnerability and central consequences of auditory deafferentation.

Previous studies of LDAEP and repetition suppression have argued that abnormalities in these measures are linked to alterations in particular neuromodulator or neurotransmitter systems. LDAEP in particular is often described as a measure of cortical serotonergic activity, based on results from pharmacological studies linking increased level dependence of the N1/P2 waves with decreased serotonergic neurotransmission [46, 47]. However, both LDAEP and repetition suppression (interval-dependent AEP, sometimes called time-dependent AEP) are blunted by administration of N-methyl-D-aspartate (NMDA) receptor blockers [48], indicating that the neuropharmacology of these AEP measures may be complex. Moreover, cortical AEPs are shaped by integration, adaptation, and synchrony of neural population activity in multiple brain areas along the ascending auditory pathway, so it is not necessarily reasonable to expect a simple one-to-one relationship between specific AEP abnormalities and particular neuromodulator or neurotransmitter systems. We suggest that systems-level mechanistic explanations for AEP abnormalities, such as increased central auditory excitability and reduced adaptation at longer time scales, are more likely to be translationally useful.

### Conclusions and future directions

We found multiple abnormalities in cortical auditory evoked potentials (AEPs) in the *Df1/+* mouse model of 22q11.2DS, some of which were strongly influenced by hearing impairment, a common co-morbidity of 22q11.2DS. *Df1/+* mice exhibited 3 key AEP abnormalities: (1) increased central auditory gain, which was especially pronounced for stimulation of ears with hearing impairment; (2) increased growth of AEP amplitude with sound level, regardless of the presence or absence of hearing impairment in the stimulated ear; and (3) increased growth of AEP amplitude with inter-stimulus-interval duration when hearing was normal in the stimulated ear. Based on these results, we suggest that level-dependent AEP growth may be especially useful as a biomarker for auditory brain abnormalities in 22q11.2DS, because this measure was robust to hearing impairment in the stimulated ear.

More generally, we conclude that auditory deafferentation and genetic risk for 22q11.2DS can have both independent and interactive effects on evoked-potential measures of auditory brain function. Further experiments are needed to address three limitations of the present work. First, a more complete dissociation of effects of genetic risk and hearing sensitivity could be achieved if the group comparisons included WT mice with induced hearing impairment similar in severity and developmental time course to that caused by otitis media in *Df1/+* mice. Second, the use of ketamine as the anesthetic could have affected the AEP results by blocking NMDA receptors [48, 49]; this potential confound might be avoided in the future by recording in awake mice. Third, and most importantly, our results were obtained in a mouse model of 22q11.2DS. Future studies in humans will be required to test the hypothesis that co-morbid hearing impairment could be a “second-hit” risk factor for brain abnormalities and psychiatric disease in 22q11.2DS patients.

### Other information

Links to references for Fig. 1: #01 [50]; #02 [51]; #03 [52]; #04 [53]; #05 [54]; #06 [55]; #07 [56]; #08 [57]; #09 [58]; #10 [59]; #11 [60]; #12 [61]; #13 [62]; #14 [63]; #15 [26]; #16 [25]; #17 [24]; #18 [64]; #19 [65]; #20 [27]; #21 [66]; #22 [29]; #23 [67]; #24 [68]; #25 [69]; #26 [70]; #27 [71]; #28 [72]; #29 [73]; #30 [74]; #31 [75]; #32 [76]; #33 [77]; #34 [78]; #35 [79]; #36 [80]; #37 [81]; #38 [82]; #39 [83]; #40 [84]; #41 [85]; #42 [86]; #43 [87]; #44 [88]; #45 [89]; #46 [90]; #47 [91]; #48 [92]; #49 [93]; #50 [94].

## Supplementary information

Supplementary Information

## Data Availability

Analysis datasets are available from the corresponding author on request.

## Code availability

Analysis code is available from the corresponding author on request.
