# Supplementary material for: Auditory evoked-potential abnormalities in a mouse model of 22q11.2 Deletion Syndrome and their interactions with hearing impairment
Source: Transl Psychiatry. 2025 Jan 8;15:4. doi: 10.1038/s41398-024-03218-x (PMC11711659; doi:10.1038/s41398-024-03218-x)
Supplement: Supplementary file 1 — Supplementary Information [file 41398_2024_3218_MOESM1_ESM.pdf]

Supplementary Information

Methods and Materials

Acoustic stimulation

Auditory stimuli were generated at a sample rate of 195,312.5 Hz using a digital signal processor (Tucker Davis Technologies, TDT RX6), attenuated as needed (TDT PA5), amplified (TDT SA1), and presented using a free-field speaker (TDT FF1) positioned 10 cm from the ear directed toward the speaker. Speaker output was calibrated to within 1.5 dB of target values before each set of experiments using a G.R.A.S. free-field 1/4" microphone, placed at the location of the ear to be tested.

Experimental procedures

Animals were anesthetized with intraperitoneally injected ketamine (70-100 mg/kg) and medetomidine (0.24-0.50 mg/kg) for recording, and were also given subcutaneous doses of carprofen (4.8-8.3 mg/kg) for analgesia and atropine (0.10-0.16 mg/kg) to minimize respiratory secretions. Supplementary doses of ketamine or ketamine/medetomidine mixture were administered if a toe-pinch response or whisker twitching was observed. Body temperature was maintained at 37-38°C using a homeothermic blanket (Harvard Apparatus). Each mouse was placed on an elevated platform and oriented with the tested ear directed toward the speaker. The opposite ear was blocked with an earplug during the recording to ensure monaural stimulation.

Subdermal electrodes (Rochester Medical) were inserted at the vertex; the bulla of the tested ear; the ipsilateral and contralateral auditory cortex relative to the tested ear; and the olfactory bulb (ground electrode). Data was acquired at a 24,414 Hz sample rate (TDT RX5) using a low-impedance headstage and signal amplifier (TDT RA4LI and RA16SD, 20x gain overall, 2.2 Hz - 7.5 kHz filtering) along with a custom low-pass filter designed to remove attenuation switching transients (100 kHz cutoff). Stimulus presentation and data acquisition was controlled using software from TDT (Brainware) and custom software written in MATLAB.

Data pre-processing

To check the quality of AEP recordings, distributions of root mean square (RMS) values of the 1000 trials in every AEP recording session were checked. No clear outlier trials that were discrete from the rest of RMS distribution were identified for any of the recordings; therefore, we didn't exclude any trials from the analysis. Three key deflections of the AEP waveform

(P1, N1, and P2) were manually selected from the averaged waveform of all 1000 trials after removing heartbeat noise [1]. Heartbeats were identified as large differences between the detrended waveform and a detrended, smoothed waveform, and eliminated by subtracting these differences from the detrended waveform.

Across all recordings from a stimulated ear and the contralateral auditory cortex, 4 recordings were excluded because no clear P1, N1 and P2 could be identified, and 2 were excluded because the mice died from possible anaesthetic overdose during recording.

References

1 de Cheveigné A. Sparse time artifact removal. *J Neurosci Methods* 2016; **262**: 14–20.

Supplementary Figures

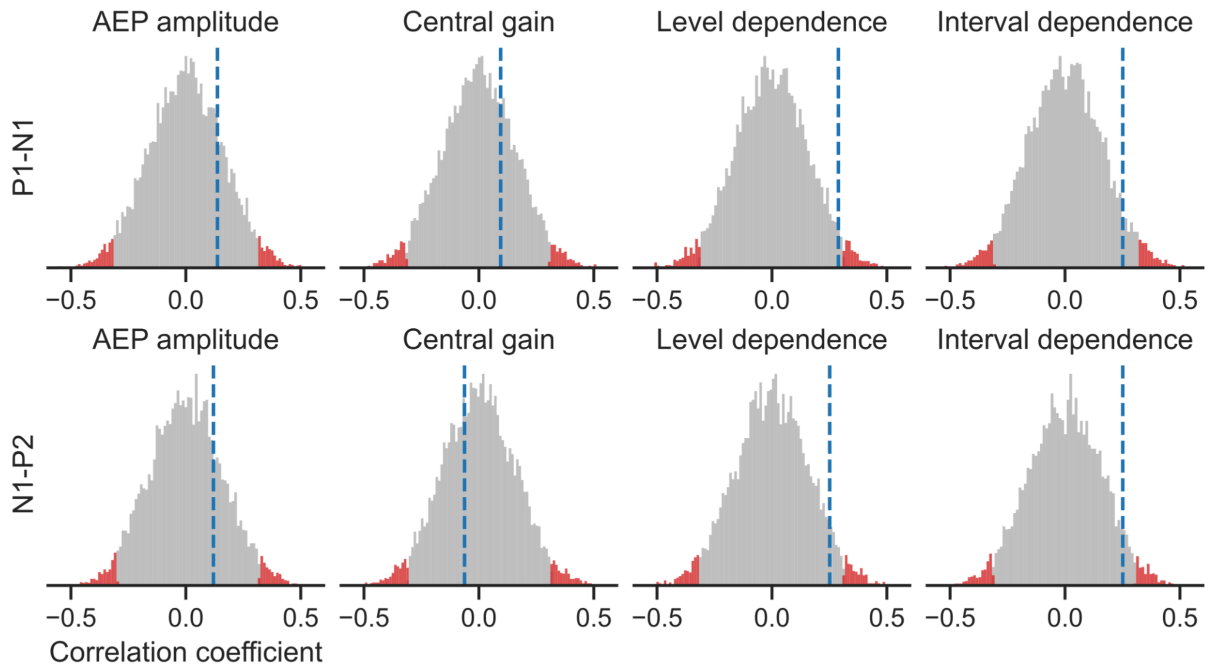

Supplementary Figure 1. Correlation between paired AEP measures from stimulation of different ears in the same animal was not significantly different from correlation between independent AEP measures from different animals. For most animals, we had two sets of AEP measures, obtained from recordings over the contralateral auditory cortex during monaural stimulation of each ear. To determine whether correlation between paired AEP measures was significantly different from that expected for independent AEP measures, we calculated Spearman's rank correlation coefficient for the paired data (blue dashed vertical line in each plot) and compared it to the distribution of correlation coefficients obtained in 10,000 randomizations of the pairing to different animals (gray histograms; lowest 2.5% and highest 2.5% of correlation coefficients highlighted in red). Within-animal correlation in AEP measures fell within the 95% confidence interval for between-animal correlation for both P1-N1 and N1-P2 waves (rows) and for all AEP measures (columns).

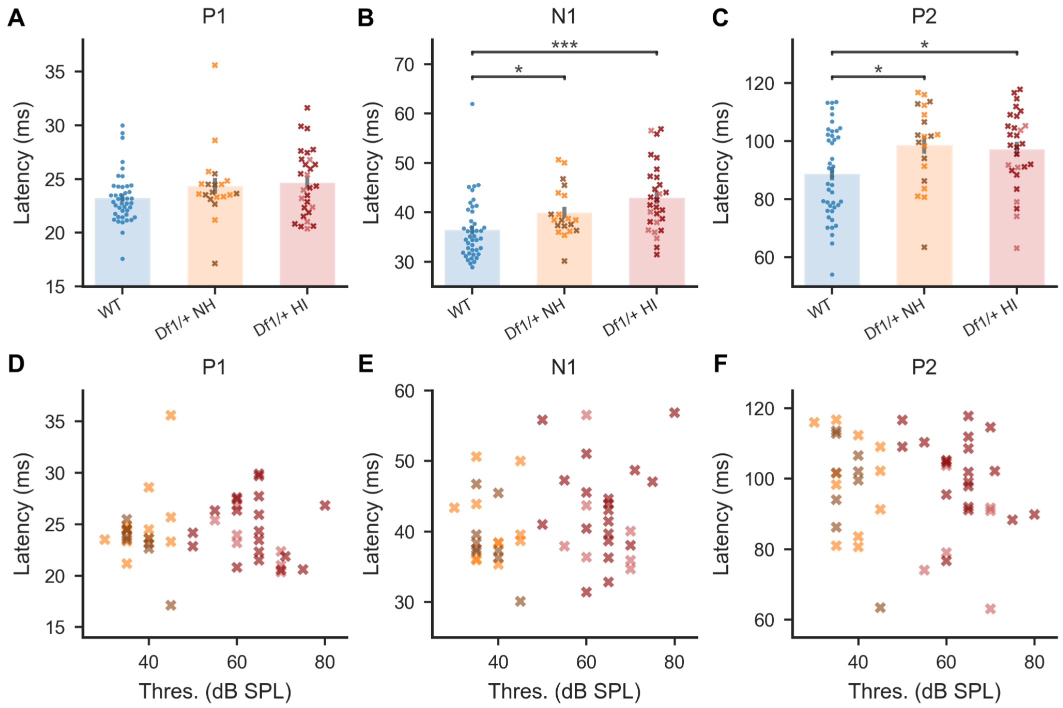

Supplementary Figure 2. Latencies of AEP waves N1 and P2 evoked by a fixed-intensity tone were abnormally elevated in *Df1*/+ mice and uncorrelated with click-evoked ABR threshold in the stimulated ear. (A-C) A fixed-intensity tone (80 dB SPL, 16 kHz) evoked longer latency N1 and P2 waves in *Df1/+* mice than WT mice, regardless of whether the stimulated *Df1/+* ear had a normal or impaired hearing threshold (B,C; N1: *Df1/+* NH 39.96 ± 5.05 ms, *Df1/+* HI 42.98 ± 6.82 ms, WT 36.39 ± 6.04 ms, $p < 0.001$, post-hoc tests, $p_{Df1/+\ NH\text{-}Df1/+\ HI} = 0.23$, $p_{Df1/+\ NH\text{-}WT} = 0.020$, $p_{Df1/+\ HI\text{-}WT} < 0.001$; P2:, *Df1/+* NH 98.64 ± 13.75 ms, *Df1/+* HI 97.25 ± 13.28 ms, WT 88.74 ± 15.09 ms, $p = 0.015$, post-hoc tests, $p_{Df1/+\ NH\text{-}Df1/+\ HI} = 0.70$, $p_{Df1/+\ NH\text{-}WT} = 0.042$, $p_{Df1/+\ HI\text{-}WT} = 0.046$). There were no significant differences between WT and *Df1/+* data for latency of the P1 wave (A; P1: *Df1/+* NH 24.35 ± 3.31 ms, *Df1/+* HI 24.66 ± 3.05 ms, WT 23.24 ± 2.33 ms, Kruskal-Wallis test, $p = 0.057$). (D-F) No significant correlations between the latency of AEP waves evoked by an 80 dB SPL, 16 kHz tone and the click-evoked ABR threshold of the stimulated *Df1/+* ear (Spearman's rank correlation tests, P1, N1 and P2 all $p > 0.05$). Plot conventions as in Figure 3.

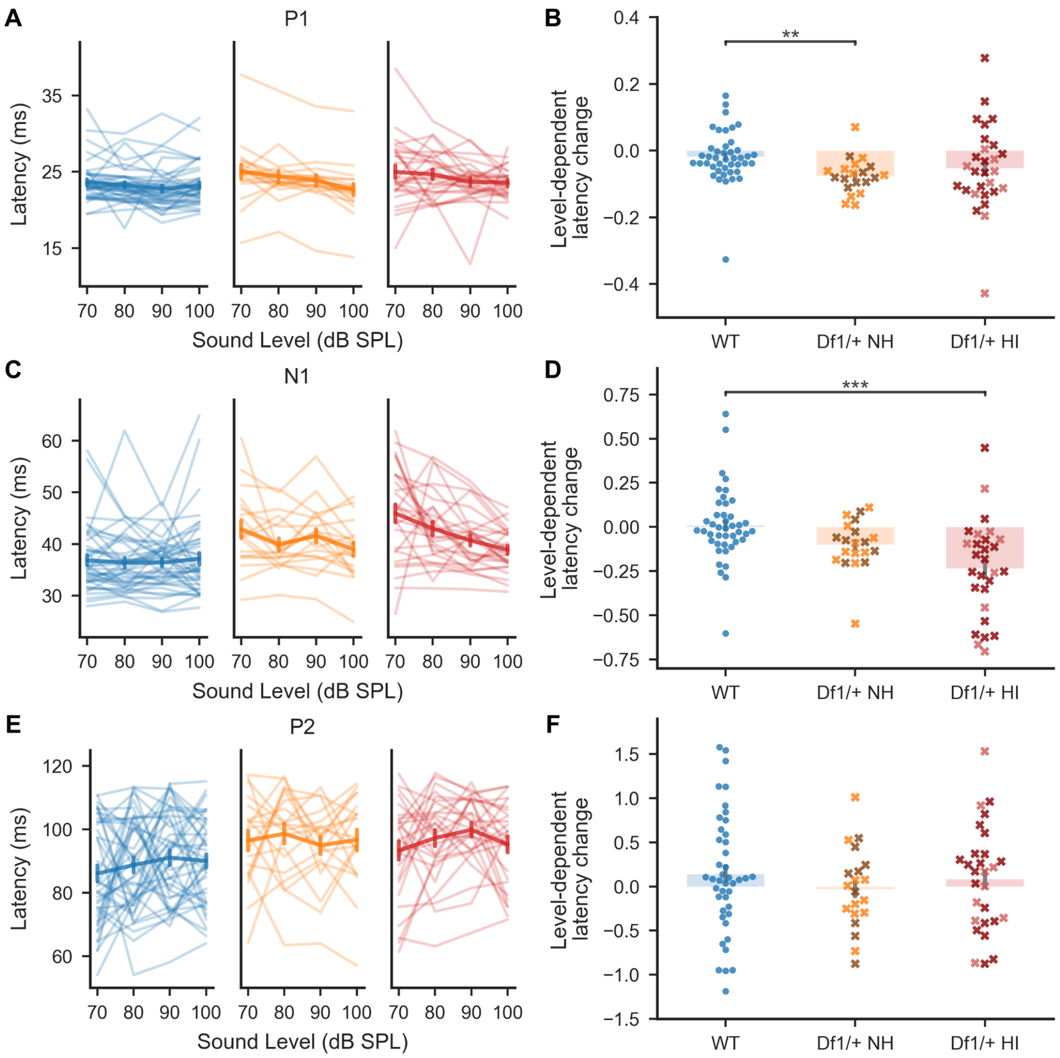

Supplementary Figure 3. Decrease of AEP wave latency with increasing tone intensity was slightly more pronounced in *Df1/+* mice than WT mice. (A,C,E) Latency for the P1 (A) and N1 (C) AEP waves evoked by a 16 kHz tone dropped slightly with increasing sound level, while latency for the P2 wave (E) was stable across sound levels in all groups (P1: Kruskal-Wallis test, p = 0.0017, post-hoc tests, $p_{Df1/+\ NH\text{-}Df1/+\ HI} = 0.18$, $p_{Df1/+\ NH\text{-}WT} = 0.0019$, $p_{Df1/+\ HI\text{-}WT} = 0.059$; N1: Kruskal-Wallis test, p < 0.001, post-hoc tests, $p_{Df1/+\ NH\text{-}Df1/+\ HI} = 0.090$, $p_{Df1/+\ NH\text{-}WT} = 0.072$, $p_{Df1/+\ HI\text{-}WT} < 0.001$; P2: Kruskal-Wallis test, p = 0.61). (B,D,F) Slopes of level-dependent latency growth functions for P1 (B) and N1 (D) were slightly steeper in *Df1/+* ears, but the growth function for P2 didn’t show significant between-group differences (F). Plot conventions as in Figure 4.

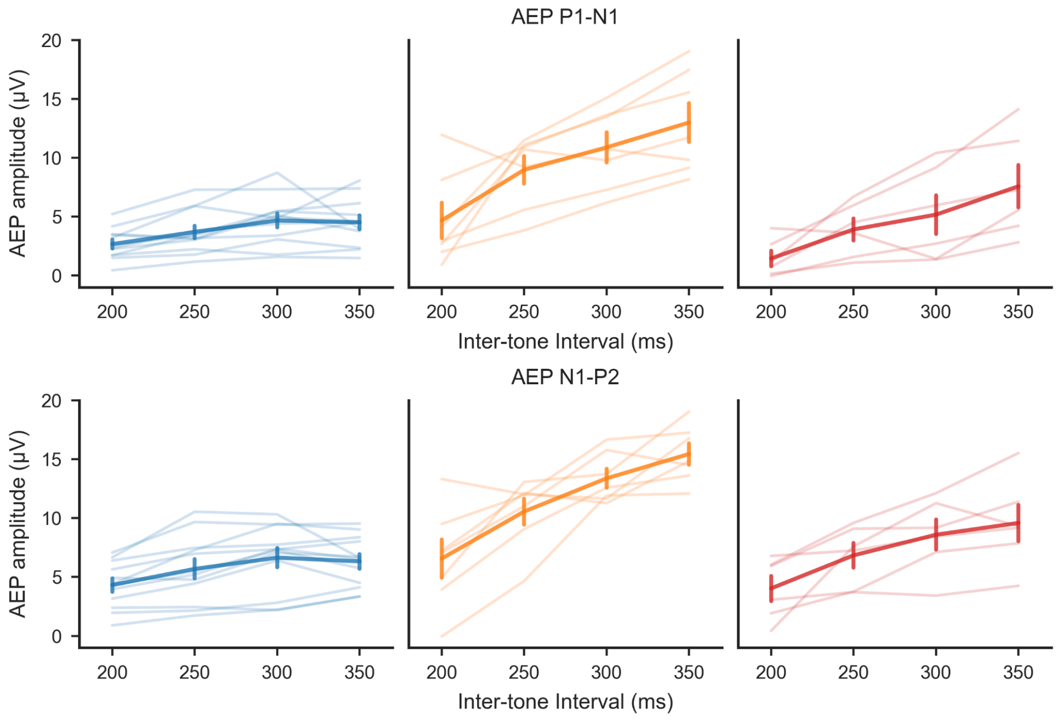

Supplementary Figure 4. Plateau in growth of AEP wave amplitude at 300-350 ms inter-tone intervals (ITIs) for WT but not *Df1*/+ mice. For both P1-N1 and N1-P2 complexes, AEP amplitude showed a plateau at longer ITIs (300-350 ms) in WT mice. In contrast, AEP wave amplitude kept increasing over this ITI interval in *Df1*/+ mice, for stimulation of both *Df1*/+ NH and *Df1*/+ HI ears. Solid lines represent AEP recordings contralateral to the stimulated ears; thick line with error bars shows mean ± SEM.

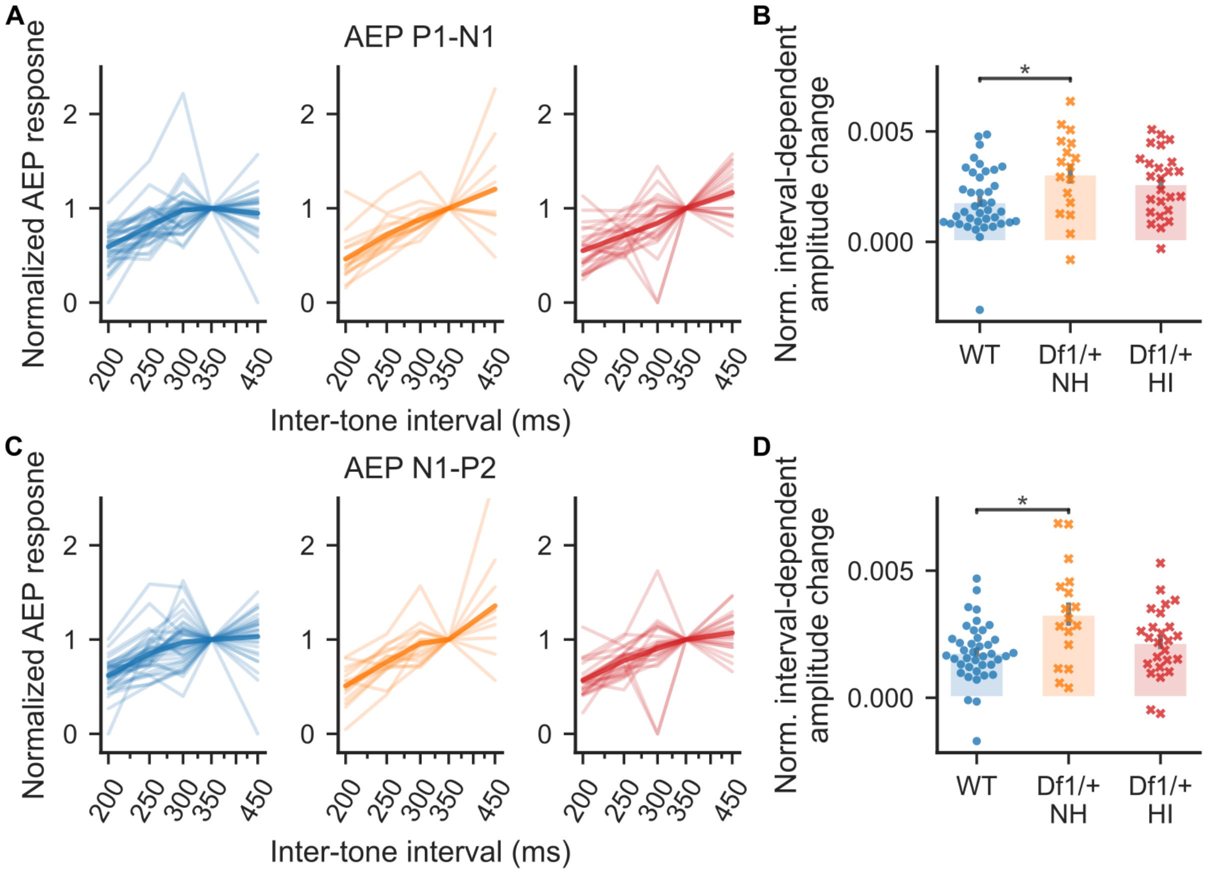

Supplementary Figure 5. Abnormalities in repetition suppression in *Df1/+* mice were also evident in normalized data. As in Figure 5C-F, plots illustrate growth of AEP amplitudes evoked by 80 dB SPL, 16 kHz tones presented at different ITIs; however, here those values are shown normalized to AEP amplitude at 350-ms ITI. (A,C) Normalized AEP amplitudes for the tone-evoked P1-N1 complex (A) and N1-P2 complex (C) grew more steeply with increasing ITI for stimulation of *Df1/+* NH ears than WT NH ears. (B,D) Normalized interval-dependent AEP amplitude change, a normalized measure of release from repetition suppression with increasing ITI, was abnormally high for stimulation of *Df1/+* NH ears. Plot conventions as in Figure 5.

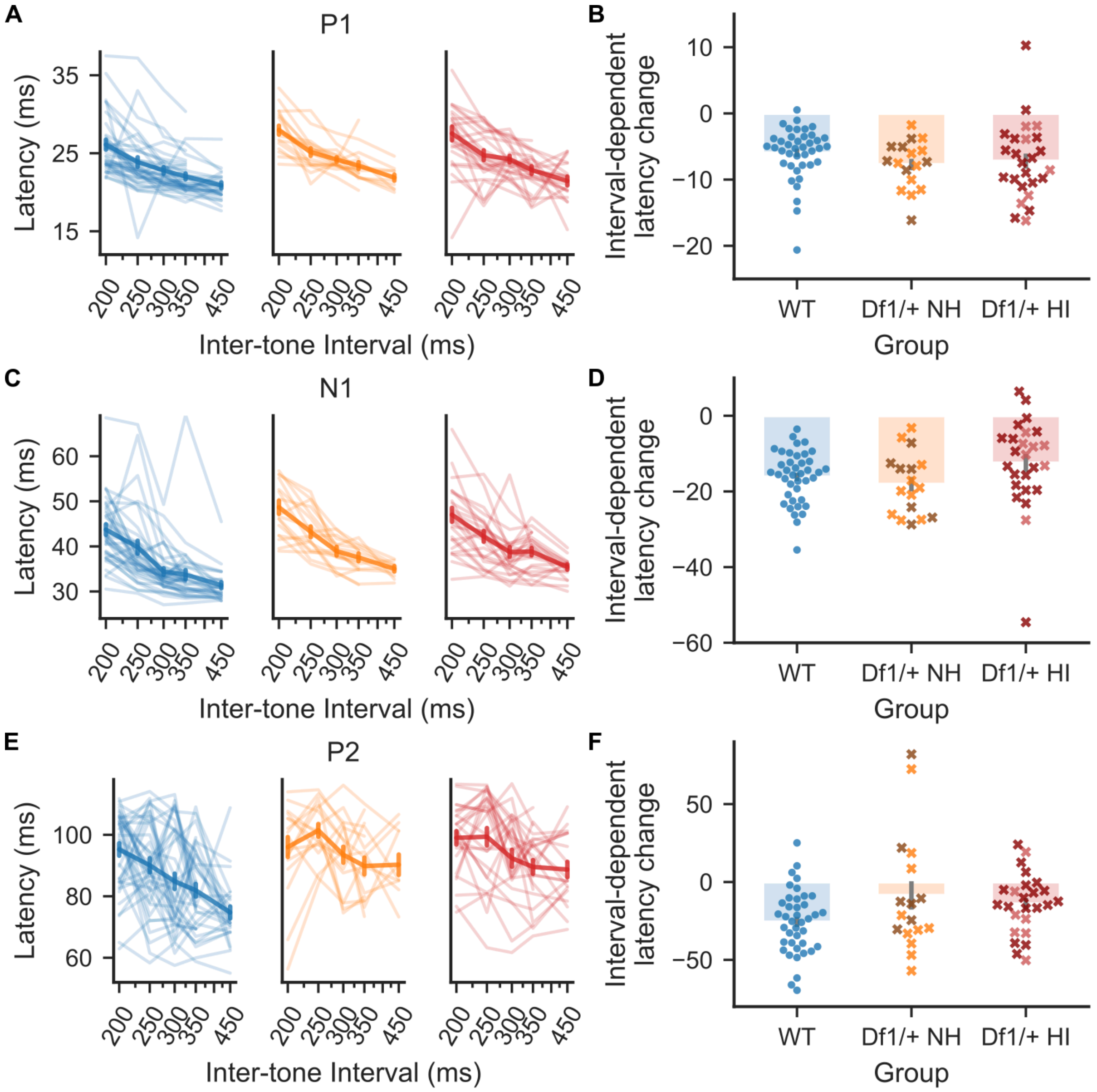

Supplementary Figure 6. No differences between *Df1/+* and WT mice in the relationship between AEP wave latency and ITI for a fixed-intensity tone. (A,C,E) Latencies of the P1 (A), N1 (C), and P2 (E) waves evoked by an 80 dB SPL, 16 kHz tone dropped with increasing ITI. Note that although AEP wave latencies were slightly elevated overall for both *Df1/+* NH and *Df1/+* HI data compared to WT data (see also Supplementary Figure 1), the relationship between wave latency and ITI was generally consistent across the three groups. Slopes of the interval-dependent latency growth functions were not significantly different between groups for P1 or P2, and were different for N1 only overall, not in post-hoc tests (Kruskal-Wallis test, P1: p = 0.11; N1: p = 0.040, post-hoc tests, $p_{Df1/+\ NH\text{-}Df1/+\ HI}$ = 0.051, $p_{Df1/+\ NH\text{-}WT}$ = 0.39, $p_{Df1/+\ HI\text{-}WT}$ = 0.10; P2: p = 0.086). Plot conventions as in Figure 5.
